# Selecting methods for draft GEM generation in multicellular eukaryotes: a comparative analysis

**DOI:** 10.1186/s12859-026-06455-7

**Published:** 2026-05-22

**Authors:** Natalia E. Jiménez, Mikael Espinoza, Sebastián Mejías, Sebastián N. Mendoza, Ignacia Segovia, J. Cristian Salgado, Carlos Conca, Ziomara. P. Gerdtzen

**Affiliations:** 1 Institute for Biological and Medical Engineering, Pontificia Universidad Católica de Chile, Santiago, Chile; 2 Department of Chemical and Bioprocess Engineering, School of Engineering, Pontificia Universidad Católica de Chile, Santiago, Chile; 3 Department of Chemical Engineering, Biotechnology and Materials, Faculty of Physical and Mathematical Sciences (DIQBM), University of Chile, Santiago, Chile; 4 Centre for Biotechnology and Bioengineering (CeBiB), University of Chile, Santiago, Chile; 5 Center for Mathematical Modeling (CMM), University of Chile, Santiago, Chile

**Keywords:** Genome-scale models, Automated reconstruction, Multicellular eukaryotes, Metabolic models

## Abstract

Motivated by the applications of genome-scale metabolic models (GEMs) for biological discovery and metabolic engineering, several approaches have been developed for automatic generation of draft GEMs. However, most of these methods are not optimized for their use in multicellular eukaryotes and their performance for this task is unclear. In this work we present a comparative analysis of seven automated reconstruction tools (AuReMe, CarveMe, merlin, ModelSEED, Pathway Tools, RAVEN and Reconstructor) applied to three multicellular eukaryotes: the mosquito *Aedes aegypti*, the CHO (Chinese Hamster Ovary) cell line from *Cricetulus griseus* and the brown algae *Ectocarpus siliculosus*. Evaluation of these tools was based on metrics for network size, functionality, consistency, representation of organelle-specific functions and organism-specific metabolites, annotation quality and execution time. We find that methods differ strongly in a trade-off between model functionality and representation of eukaryotic features such as compartmentalization and organism-specific metabolism, with no single approach excelling at both. Our work aims at providing a practical resource to guide researchers in selecting methods for draft generation tailored to organism characteristics and research goals.

**Supplementary Information:**

The online version contains supplementary material available at 10.1186/s12859-026-06455-7.

## Background

Genome-scale metabolic models (GEMs) are formal representations of the biochemical reactions that can take place in organisms and their requirements on genes and proteins, inferred from annotated genomes [1, 2]. GEMs have emerged as a powerful tool for biological discovery and metabolic engineering, due to their ability to simulate gene knockouts [3–6], describe multi-species relationships [7, 8], contextualize omics data [9, 10], identify gene targets for cancer therapy [11, 12], and provide a framework for mechanistic understanding of phenotypes of interest [13].

The process of manually generating GEMs is complex and time-consuming, often taking up to several years [2]. This fact, alongside the explosion of genomic data driven by modern sequencing technologies [14–17], motivated the development of several automatic strategies for generating draft GEMs from sequenced genomes [18]. These automated tools, rely on specialized biochemical databases (Reconstructor [19], AutoKEGGRef [20], RAVEN 2.0 [21]), their own genome annotation (merlin [22]) or a curated available version including EC numbers to establish a draft version of the desired GEM (Pathway Tools [23], CarveMe [24], ModelSEED, PlantSEED [25, 26], gapseq [27]). Orthology inferences can also be used to retrieve candidate reactions (AuReMe [28]) and information from previously curated models can aid in this process (AuReMe [28], Reconstructor [19], RAVEN2.0 [21]).

While these automated approaches have been successfully applied for generating draft GEMs, their primary focus has been on prokaryotes and unicellular eukaryotes such as *S. cerevisiae* [29] and *Y. lipolytica* [30]. There are fewer examples of their use in multicellular eukaryotes, with some exceptions such as PlantSEED (based on ModelSEED) and the use of these strategies for generating plant models [22, 25], macroalgae [31] and mammalian cell lines [32]. Automatic drafting of eukaryotic metabolism poses additional challenges related to genome size and complexity [33, 34], intracellular gene location [35, 36], as well as harboring information regarding different cellular types that can display varied metabolic behaviors [37].

Relative performances of different automated reconstruction tools have not been systematically assessed for multicellular eukaryotes. Furthermore, it has not been assessed which model metrics would be relevant to compare for applications of multicellular eukaryotic GEMs. This is a critical issue as metabolic understanding of these organisms has deep implications for public health [38], sustainable economy [31] and the biopharmaceutical industry [32].

In this work, we provide a perspective on the automatic draft GEM reconstruction methods for modeling multicellular eukaryotes. Three organisms with different degrees of prior metabolic characterization were chosen for this comparison: (i) the Chinese Hamster Ovary (CHO) cell line from *Cricetulus griseus*, which has an extensively curated GEM widely used for basic and applied metabolic research [39–41], (ii) the brown algae *Ectocarpus siliculosus* whose interactions with its microbial communities have been studied using GEMs [42], and (iii) the mosquito *Aedes aegypti*, which has a reconstruction used for studying its interactions with its endosymbiont *Wolbachia pipientis* [38, 43]. In this study, we provide a thorough analysis of the most relevant automated tools for generation of draft GEMs compatible with eukaryotic organisms, highlighting relevant challenges that arise when working with such organisms.

## Materials and methods

### Draft generation

Three organisms from a wide range of phylogenetic groups and different degrees of knowledge were selected to make this evaluation: (i) *Cricetulus griseus* and its derived cell line CHO (Chinese Hamster Ovary), for which there is a highly curated genome and GEM [32] and has been widely used in different applications [44, 45], (ii) *Ectocarpus siliculosus*, a brown kelp for which there is a genome-scale model [31], and (iii) *Aedes aegypti*, a mosquito for which there is a recent metabolic reconstruction [38].

*Cricetulus griseus* genome (CriGri_1.0) [46] and its annotation were retrieved from NCBI (GCA_000223135.1). *A. aegypti* genome (AaegL5.0) [47] and its annotation were retrieved from VectorBase. *E. siliculosus* genome (ASM31002v1) [48] and its annotation were retrieved from NCBI (GCA_000310025.1).

Seven frameworks for generation of draft genome-scale models were retrieved based on literature and their capabilities to handle eukaryotic genomes. The process to generate draft models using these approaches is described below and summarized in Table S1.

### AuReMe

AuReMe (AUtomated REconstruction of MEtabolic models) [28] is a method for reconstruction of metabolic models which allows the incorporation of information included on a template genome-scale model. It starts from an annotated genome of the target processed through Pathway Tools [23] to obtain a draft model. This information is complemented with ortholog data that is mapped to a template model to retrieve additional reactions and gene associations to be included in their draft model. Annotation and orthology-based drafts were generated using AuReMe (version 2.4) as follows: organism-specific Pathway Tools annotation folders (.dat folder) were provided for the annotation-based step, and different reference organisms with their respective models were used for the analyzed organisms in this work. *Drosophila melanogaster* (Release 6 plus ISO1 MT, GCF_000001215.4) was used as reference organism for *A. aegypti* [49], *Mus musculus* (GRCm39, GCF_000001635.27) [50] for CHO (*C. griseus*) [51] and *Chlamydomonas reinhardtii* (Chlamydomonas_reinhardtii_v5.5, GCF_000002595.2) [52] for *E. siliculosus* [53]. Additional reconstructions were obtained using *Homo sapiens* (GRCh38.p14, GCF_000001405.40) [54] for *A. aegypti* and CHO, and *Saccharina japonica* [28, 55] for *E. siliculosus* to test the effect of reference organisms in the obtained quality of reconstructions.

### CarveMe

CarveMe [24] starts from an universal model which is carved based on information contained on its proteome. This method, originally developed from prokaryotes, proposed a biomass adapted from *E. coli*, and removes blocked reactions and dead-end metabolites to retrieve a functional model. In this work, CarveMe [24] was used with amino acid fasta files of the target organisms as input using their default settings (bacterial template and the arguments—more-sensitive—top 10 for DIAMOND [56]).

### Merlin

Merlin [22] is a java application with its own user interface that performs its own annotation, transport and compartment location predictions and retrieves a draft model that can be curated by the user. It performs enzyme annotation on its own server using BLAST or DIAMOND as selected by the user. It has been used in eukaryote organisms such as the oak tree (*Quercus suber*), and it includes modules for integration with MEMOTE [57] and Escher [58] for visualization. In this work, merlin 4.014 [22] was used, homology search was performed using DIAMOND [56] with the default e-value (1e-30) and KEGG metabolic data was loaded into their workspace. Enzyme annotation and integration were performed through their automatic workflow complemented by Transyt [59] to create transport reactions. Subsequently, the compartment report from Deeploc2 [60] was loaded and integrated into the model. Finally, the biomass equation was created using yeast as a template.

### PlantSEED

ModelSEED [4], is a web-based platform that allows users to retrieve draft, functional models from their genome. To achieve this it performs its own annotation and gapfilling with a biomass reaction based on taxonomy. An extension of this framework, plantSEED, was later introduced for studying plant primary metabolism [25], expanding their predictions to eukaryote organisms, and offering integrated tools for running Flux Balance Analysis through the same web interface. An implementation of ModelSEED for eukaryote organisms (PlantSEED) [25] was used in this work with amino acid fasta files of the target organisms studied. A ‘complete’ medium was selected to perform draft model generation, which considers presence of a metabolite in the media only if there are transporters for this metabolite in the model.

### Pathway tools

Pathway Tools [23] supports the creation and management of organism-specific Pathway/Genome Databases (PGDBs). Using an annotated genome as input, its PathoLogic module builds a new PGDB for prediction of metabolic pathways, genes and biochemical reactions. In this work, the published genome annotation was complemented with eggnogmapper [61] with their web based version using standard parameters (minimum hit e-value: 0.001, Minimum hit bit-score: 60, percentage identity: 40, minimum % of query coverage: 20, minimum % of subject coverage: 20), using Eukaryota as their taxonomic scope. Obtained results were then combined to generate gbk files using emapper2gbk [62] to retrieve gbk files compatible with Pathway Tools, which were subsequently used to generate the draft models with default parameters.

### RAVEN 2

RAVEN 2 (Reconstruction, Analysis and Visualization of mEtabolic Networks) [21] provides tools for *de novo* draft genome-scale model reconstruction using MetaCyc, KEGG, or a combination of both, relying on protein homology obtained using BLAST. It can also incorporate information from existing genome-scale models and has been applied to prokaryotes, eukaryotes, and even specific cell types. An implementation of RAVEN 2 (MATLAB 2015a) was used to generate a combined draft GEM from KEGG and MetaCyc as specified on their documentation [21]. In summary, a MetaCyc-based draft (‘raven-metacyc’ in this work) was generated from amino acid fasta files, keeping transport reactions with other parameters set as default (exclusion of unbalanced and undetermined reactions, minimum bit score: 100, minimum positive values: 45%, DIAMOND as alignment tool to perform homology search). A KEGG-draft was generated based on sequence homology (‘raven-kegg’) of fasta amino acids file to KEGG Ortholog sequence clusters, incorporating the eukaryote cluster “euk90_kegg105”, while excluding incomplete reactions and the ones with undefined stoichiometry, the other parameters are set as default (cutOff: 1e-50, minScoreRatioG: 0.8, minScoreRatioKO: 0.3, seqIdentity = 0.9).

Finally, both draft models were combined into an integrated GEM (‘raven-comb’) using the function combineMetaCycKEGGModels, which converts metabolite and reaction identifiers in the KEGG model into corresponding MetaCyc IDs, and then detects duplications and keeps only unique reactions and metabolites that are mostly in the MetaCyc namespace.

### Reconstructor

Reconstructor is a COBRApy-compatible method used for generation of draft genome-scale models [19]. It starts from amino acid fasta files, which are aligned to the KEGG database using DIAMOND [56]. Results are then conciliated with the ModelSEED database [26] to suggest reactions to be included in the draft model. In this work, Reconstructor [19] was used with amino acid fasta files of the target organisms as input, using default parameters (minimum objective fraction required during gap filling: 0.01, maximum objective fraction allowed during gap filling: 0.5) with a Gram-negative classification.

### Analysis of draft models

Different metrics were selected to assess features of the obtained draft models. MEMOTE [57] was used to compute the number of genes, reactions, metabolites, compartments, gene associated reactions (enzymatic reactions), reactions able to carry flux in complete medium (flux reactions), annotation score and consistency score of the obtained draft models. The annotation score reflects the extent to which model annotations comply with the MIRIAM (minimum information requested in the annotation of biochemical models) guidelines [63], use a unified namespace, and include Systems Biology Ontology (SBO) terms [57]. The consistency score reflects stoichiometric, charge, mass, flux, and thermodynamic consistency [57]. Execution times were logged manually for their analysis.

### Metabolite, reaction and gene set comparisons

Metabolites and reactions from different namespaces were translated to MetaNetX namespace [64] for comparing model contents. Two different mapping strategies were used: (i) direct mapping via the MetaNetX database, after removing compartment-related suffixes for deduplication of biochemically equivalent reactions. This procedure was complemented with information from BiGG [65], ModelSEED [26] and the KEGG [66] REST API in regard to metabolite names and chemical composition. Stoichiometry was used as additional information for reaction mapping as described previously [18]. (ii) Model mapping using Python package mergem v.1.1.0 [67], with MetaNetX as the target database. Compartment-related suffixes were removed after mergem mapping; manual curation of the obtained mappings was performed to resolve conflicting mappings between these alternative methods. Gene identifiers were mapped to the common NCBI namespace using a custom Python script and mapping tables from Phaeoexplorer [68] and VectorBase [69].

After mapping, metabolite, reaction and gene sets were compared. The agreement of reference and draft sets was quantified by Jaccard indices (the ratio between the size of the intersection and the union of two sets). To assess all-vs-all similarities and identifying sources of variation, Principal Coordinate Analysis (PCoA) and biclustered heatmaps were performed over binary representations of model contents, in which a metabolite, reaction or gene is assigned a 1 if present in the model or 0 otherwise. Graphics were generated in Python with the matplotlib and seaborn packages [70, 71].

To study the effects of reference models in AuReMe, three models were used for comparison: one based solely on annotation, and two combining annotation-based reconstructions with orthology information. The latter included the approach used in the main analyses (Materials and methods), and another employing additional reference models (*H. sapiens* [54] for *A. aegypti* and *C. griseus*, and *S. japonica* [55] for *E. siliculosus*). Results were compared at metabolite, reaction and gene levels, by extracting this information using COBRApy [72], and plotted using matplotlib and seaborn [70, 71]. Since metabolites with identifiers from different databases are present, a script (described in the “Metabolite, reaction and gene set comparisons” section) was used to ensure database consistency among obtained drafts.

### Gene-protein-reaction rule comparisons

Gene-protein-reaction (GPR) rules are strings of genes connected by logical operators (OR or AND), encoding the dependence of reactions on genes and their products. To study the agreement of these representations in draft and reference models, a similarity metric was computed which takes into account the identity of the genes participating in reaction catalysis, as well as their specific combination.

For each MetaNetX reaction ID in a model, GPRs from all reactions that were mapped to that ID were combined by OR operators, and the combined GPR was transformed into Disjunctive Normal Form (disjunct clauses, where a clause is a conjunction of genes). As biochemically equivalent reactions in different compartments are mapped to the same MetaNetX ID, this removes the influence of compartmentalization on subsequent GPR comparisons. Combined GPRs were mapped into the NCBI namespace as previously described, and represented as a set of clauses. Then, for each corresponding MetaNetX reaction between draft and reference models, a Jaccard index was computed between the set of clauses in both GPRs, as well as the average of these Jaccard indices.

To compare the presence of GPR associations between draft and reference models, we computed the Matthews Correlation Coefficient (MCC) [10.1186/s12864-019-6413-7] based on the presence or absence of GPR associations. For each pair of draft and reference models, reactions mapped to a common MetaNetX identifier and present in both models were considered. Each shared reaction was classified as enzymatic or non-enzymatic depending on whether a non-null GPR was present in the corresponding model. The MCC was calculated between the binary vectors representing GPR presence in the draft and reference models, providing a balanced measure of concordance that accounts for both agreement and disagreement in GPR assignment.

### Presence and producibility of biomass precursors

Biomass composition was retrieved from published models for *A. aegypti* [38], *C. griseus* [32] and *E. siliculosus* [31] and translated into other databases as needed (MetaCyc [73], BiGG [65], ModelSEED or KEGG [66]). Obtained biomass metabolites were categorized into Carbohydrates, Inorganic compounds, Lipids, Nucleic acids and Organic acids using MetaboAnalyst 5.0 [74] and their presence and producibility in draft models were scored according to the fraction of present metabolites and the fraction of present metabolites that are producible, respectively. Producibility was assessed via FBA simulations using COBRApy [72] with a complete medium obtained by opening all exchanges of the model and setting the production of each metabolite as the objective function.

### Metabolomic coverage analysis

Organism-specific metabolome data was retrieved from literature for *Aedes aegypti* [75], CHO cells [32, 76–84] and *E. siliculosus* [85] and mapped to the MetaNetX namespace [MetaNetX]. Metabolomic sets were constructed for each organism by assigning a single representative MetaNetX identifier (the lexicographically largest) to each query metabolite from that organism, and then compared using a Venn diagram.

Coverage of each metabolomic set by models of the corresponding organism was assessed, both for the entire set and for its organism-exclusive subset (identifiers not contained in the metabolomic sets of the remaining organisms). A MetaNetX identifier from the metabolomic set was considered covered by a model if either the identifier itself or any of its synonyms (MetaNetX identifiers that co-occurred with it during the initial mapping) was present in the model. All visualizations were generated using the seaborn [70, 71] Python library.

### Compartment analysis

Duplicated reactions were selected based on the participation of duplicated metabolites and their stoichiometry. For each duplicated reaction, gene associations were studied to determine if they were identical to the cytoplasmic form of that reaction and classified in terms of presence of GPR and their uniqueness. Plots were made using seaborn [70, 71].

## Results

### Metric selection

Eukaryotic organisms are complex, they harbor specialized metabolic pathways yet to be discovered, perform chemical transformations in organelles, and exhibit higher genome size with respect to prokaryotes, posing additional challenges for generating GEMs. An ideal method should be able to overcome these challenges and provide a draft GEM that includes prior biological knowledge from the organism of interest, represents multiple compartments whilst preserving association with organelle-specific enzymes, and that is able to synthesize as many metabolites that comprise its biomass as possible. Generated models should also include links to different databases for metabolites and enzymes to facilitate the integration with models from other databases as well as from omic data analyses [57].

**Fig. 1:**
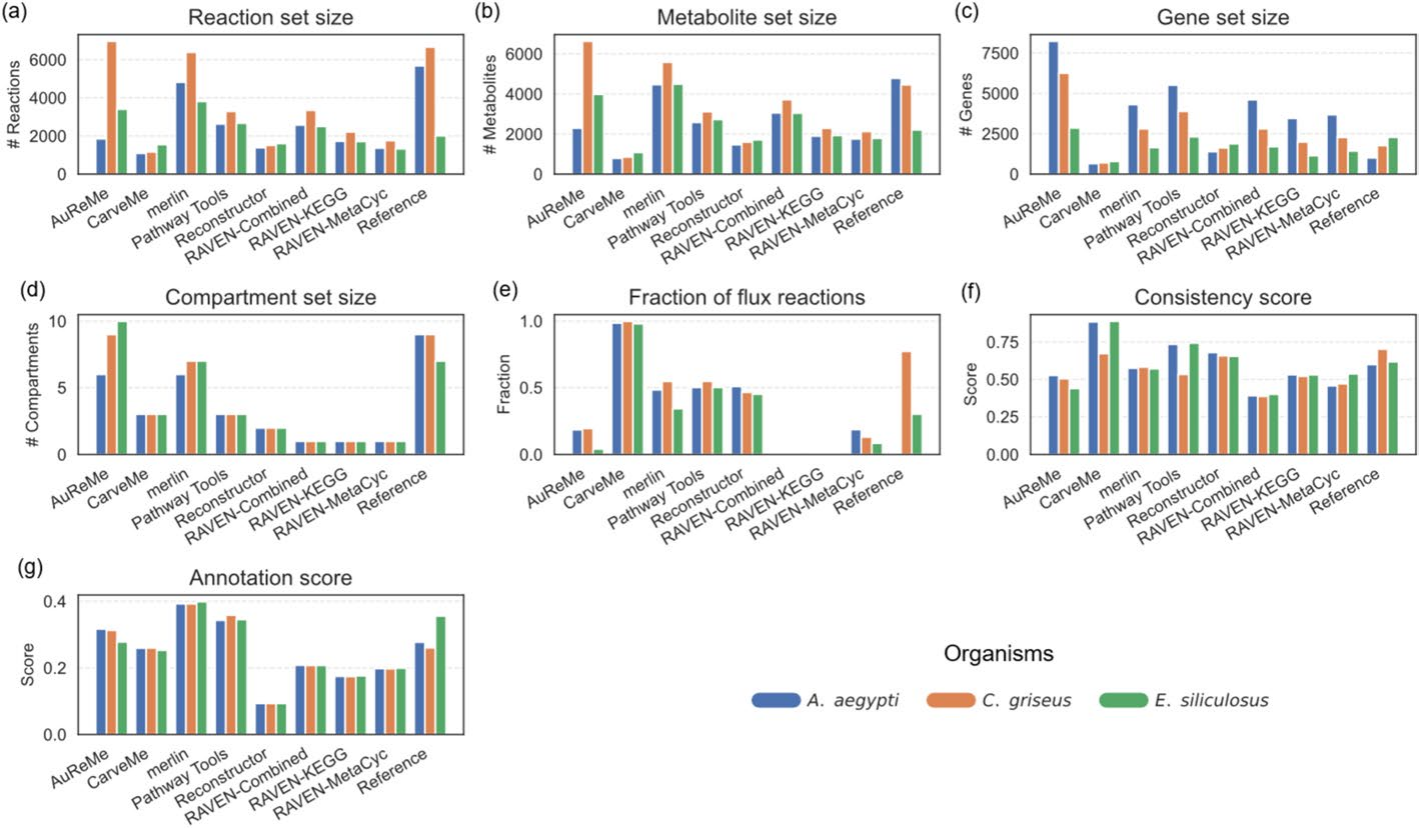
Obtained draft GEMs differences in: size (reactions (**a**), metabolites (**b**), genes (**c**), compartments (**d**)), functionality (fraction of flux reactions (**e**), consistency score (**f**)), and annotation quality (annotation score (**g**))

In this work we analyzed seven methods for generation of draft GEMs: AuReMe [28], CarveMe [24], merlin [22], PlantSEED [25], Pathway Tools [23], RAVEN [21] and Reconstructor [19]. Ten metrics were selected to characterize these methods, aligned with the desired features described previously. Selected metrics reflect the size of draft GEMs (number of reactions, metabolites, genes and compartments), how close they are to be able to produce biomass (i.e. be *f*unctional) (biomass metabolites, flux reactions, consistency), how fast is the reconstruction process (time), as well as its quality for their use in applications such as analyzing gene expression data (annotation score, enzymatic reactions) (Fig. 1, Table S2).

### Obtained drafts are influenced by design decisions of automated reconstruction tools

A basic characterization of obtained drafts was performed with MEMOTE, showing dependence on both organism and method (Fig. 1). PlantSEED models were the exception as this method was insensitive to input organism, yielding identical biochemical networks (1,179 metabolites and 1,121 reactions, Table S2) and differ only in the number of genes (1 (Unknown) *A. aegypti*, 634 *C. griseus*, 728 *E. siliculosus*) (Table S3). This behavior reflects the extensive default gap-filling performed by PlantSEED (64% of reactions are able to carry flux) which is consistent with the design described in [25] (Table S2). Because the resulting models are effectively invariant across organisms, PlantSEED outputs were excluded from further comparative figures and analyses.

Reconstructor [19], based in the ModelSEED database, showed sensitivity to organism-specific input. Although it is based in bacteria, obtained models yield on average a larger number of genes (128%), reactions (20%) and metabolites (77%) compared to other bacterial-based methods like CarveMe. However, its annotation score is considerably low (less than 0.1) being the worst in this category, which would be a disadvantage for analysis of omic datasets or cross-database comparisons, requiring extra mapping efforts.

CarveMe generates models with the highest proportion of reactions being able to carry flux (98–99%), a consequence of its top-down approach for draft generation. This method starts its reconstruction process from an universal model and it eliminates reactions for which there is no evidence of their presence [24].

AuReMe [28] incorporates information from a template model to retrieve additional reactions and associated genes based on orthology. Criteria for model selection is based on phylogenetic closeness, with the exception of *E. siliculosus*, for which a model for *S. japonica* [55] was excluded since it was based on the curated model of *E. siliculosus* [31] and a *Chlamydomonas reinhardtii* model was used instead [53]; for *A. aegypti* a *Drosophila melanogaster* model was used as a template, and for *C. griseus* a *Mus musculus* model [51] was selected to this end. Obtained draft models are highly influenced by characteristics of their corresponding templates, where inclusion of reactions and metabolites is driven by how curated each model is, rather than phylogeny alone, where bigger models that include more metabolic information (such as *H. sapiens* [54] and *S. japonica* [86]) resulting on bigger models (Table S2, File S4).

Models generated using merlin include predictions of subcellular localization of enzymes codified in their genome (deeploc2 [60]). Therefore, models generated by this tool exhibit more compartments in average than others (merlin: 7, CarveMe: 3, Reconstructor: 2, RAVEN: 1, Pathway Tools: 3, AuReMe: 6–10); thus leading to having more reactions (2894 *A. aegypti*, 6390 *C. griseus*, 3813 *E. siliculosus*). Particularly for *C. griseus*, 57% of their 5,808 metabolites are duplicated in different cellular compartments. These models also exhibit high variability in reactions that can carry flux (13 to 54%) showing an organism-dependent behavior in regard to their functionality.

Pathway Tools generated models also exhibit an organism-dependent behavior, displaying higher gene numbers in *A. aegypti* (5509) compared to *C. griseus* (3891), despite being metabolic networks with comparable size in terms of metabolites and reactions. *E. siliculosus* exhibits more reactions than genes, which could be due to its automated gap filling, and also to biological properties to this macroalgae which is unable to grow axenically [23]. This tool also includes generic reactions that must be furtherly curated by the user of the model, and that can generate issues in Flux Balance Analysis simulations for polymerization reactions if ignored.

RAVEN 2 [21] allows generating draft GEMs using different approaches: a MetaCyc-based draft (‘RAVEN-MetaCyc’ in this work) generated from amino acid fasta files, a KEGG-based draft based on sequence homology (‘RAVEN-KEGG’), as well as a combination of both approaches (‘RAVEN-Combined’). RAVEN represents a single compartment and uses a conservative approach where only reactions with gene associations are included. Due to the resulting metabolic gaps, these models exhibit a lower proportion of reactions able to carry flux (8% *E. siliculosus* to 18% *A. aegypti*). Particularly, in models generated using RAVEN linked with the KEGG database, no reactions are able to carry flux in all models due to the lack of exchange reactions.

Time required to obtain draft reconstructions varied greatly across evaluated tools, ranging from minutes to days. Merlin had the longest processing time with draft reconstruction taking up to five days (Table S2). This delay is primarily due to the BLAST performance on their servers as well as the use of external applications such as deeploc2 and the constant input from the user is required and contributes to longer processing times. On the other hand, CarveMe was found to have the shortest processing time taking up to 5 min, whereas AuReMe exhibited changes in time depending on the selected template, ranging from 20 min to 5 h.

Size of the obtained metabolic networks (number of reactions and metabolites) is highly influenced by information available on databases used by each method. This is more evident in the case of *C. griseus* models, which being closer to *H. sapiens* are reconstructed using information from this organism yielding models with over 6000 reactions (6968 AuReMe, 6390 merlin), where *A. aegypti* and *E. siliculosus* exhibit values near 3000 reactions (3813 merlin, 3403 AuReMe).

Organism-dependent functionality analysis hints issues with boundary reaction definition in draft models.

**Fig. 2:**
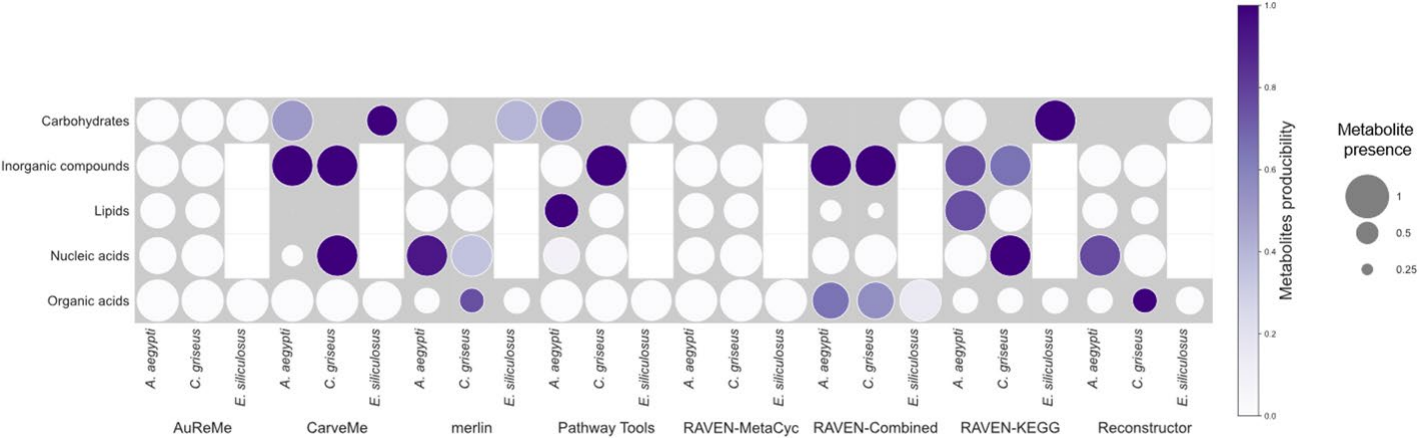
Presence and producibility of organism-specific biomass metabolites for obtained draft models. Fraction of present metabolites of a given category is depicted by circle size. Fraction of present metabolites that are also producible using complete media is depicted by circle color. White rectangles indicate categories that are not present in their respective reference organism-specific biomass

Given the relevance of biomass synthesis in GEMs [87], draft models were studied in terms of how closely they represent organism-specific curated versions of their biomass, rather than proposed biomass reactions from their respective methods. To achieve this, presence and producibility of biomass metabolites from reference models (Figure S5) was assessed in obtained drafts (Fig. 2), finding both organism and method-dependent issues in the functionality of these models.

*E. siliculosus* curated GEM presents a simpler representation of its biomass comprising organic acids (mostly amino acids, together with carboxylic acids) and carbohydrates (glycerol, mannitol, glycollate, glycerate and threo-ds-iso-citrate) [31], whereas *A. aegypti* and *C. griseus* exhibit more complex biomass representations including lipids, nucleic acids and inorganic compounds (Figure S5). Only *C. griseus* included more than one biomass function, representing a mammalian cell line producing a recombinant protein which only affected its stoichiometric coefficients.

Inorganic compounds are ions and metals that are directly imported into the cell from their surrounding media, their lack of producibility on certain methods (AuReMe, Pathway Tools) hints issues with the definition of exchange reactions in draft models. These reactions represent boundary conditions of the surroundings of the modeled organisms. In fact, despite including metabolites from their biomass for all three organisms, models generated with Pathway Tools are unable to produce them in complete media, for which manual curation is required to obtain functional models with this approach.

On the other hand, draft models obtained using RAVEN-MetaCyc, RAVEN-KEGG, RAVEN-Combined and Reconstructor can import inorganic compounds, and hence integrate them to their biomass. Reconstructor models are also able to produce nearly 60% of the organic acids present on their drafts, and together with CarveMe are the only ones able to synthesize nucleic acids in complete media. RAVEN-MetaCyc and RAVEN-Combined exhibit producibility of organic acids, but not for the remaining analyzed categories, hence not retrieving functional models.

Consistent with the top-down approach performed by CarveMe, models generated with this tool exhibit the highest producibility of biomass metabolites. These draft models exhibit a reduced capability of production for nucleic acids in *A. aegypti* (50%, 4) and carbohydrates for *E. siliculosus* (75%, 3). However, no lipids were mapped into these draft models of the 6 and 11 present in *A. aegypti* and *C. griseus* biomass respectively, showing a potential database dependency on the presence of metabolites included in obtained draft models using these tools.

Drafts differ strongly in their ability to recover eukaryotic compartmentalization.

**Fig. 3:**
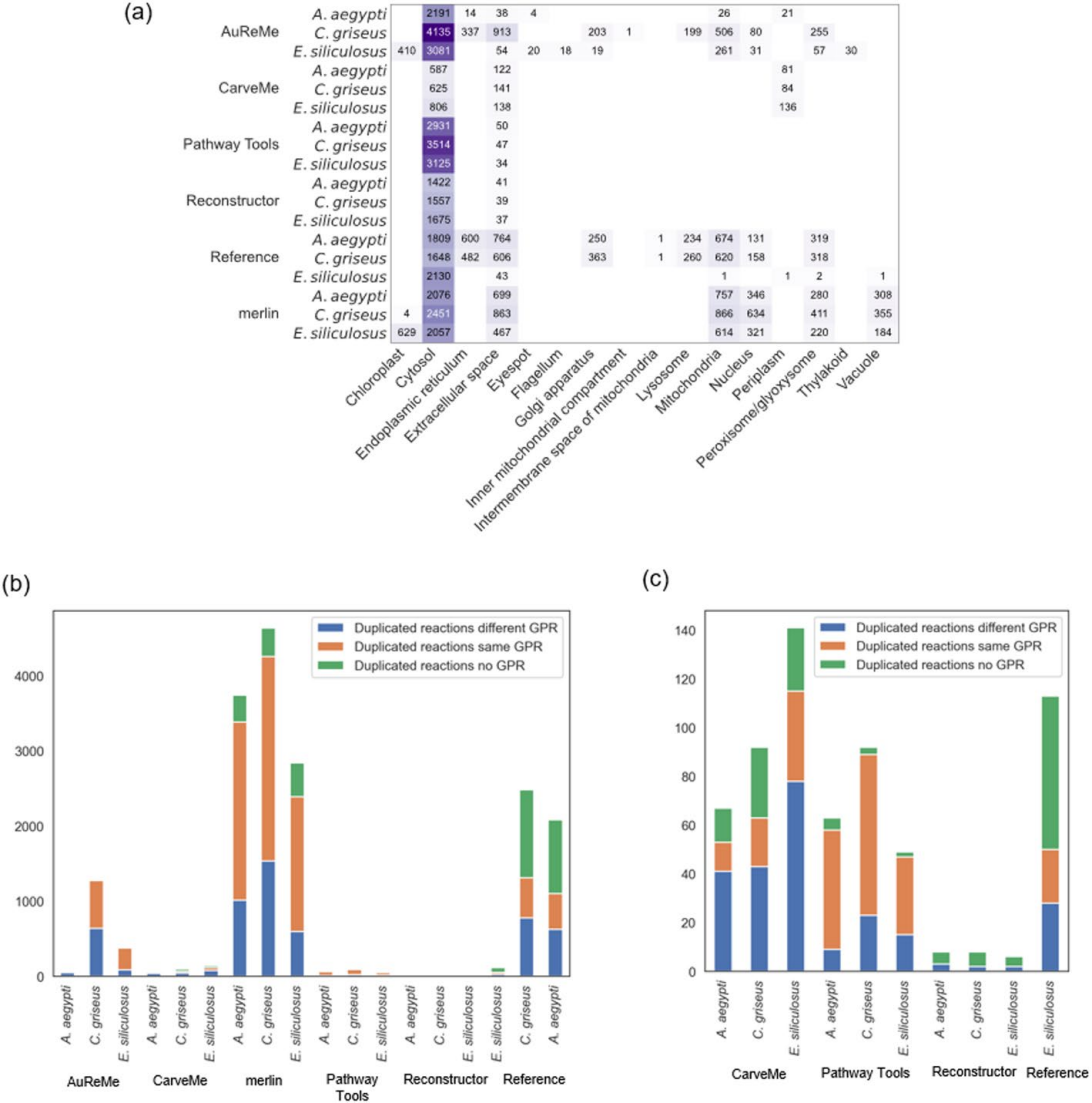
Compartmentalization analysis. Metabolites associated with different compartments for obtained drafts (**a**). Duplicated reactions in different compartments were analyzed to check if they displayed gene associations, and if these are duplicated to their cytosolic counterpart (**b**), a zoomed in section is displayed for models with fewer duplicated reactions (**c**)

Eukaryotic organisms are highly compartmentalized thus having specific functions that are encoded by different enzymes. This results in bigger metabolic networks which include multiple versions of reactions in different compartments that are usually linked to different genes in their GPRs. We aimed to assess how different frameworks for GEMs reconstruction can incorporate this information on their draft models (Fig. 3).

*A. aegypti* and *C. griseus* reference models exhibit 8 compartments with at least 100 metabolites (cytosol, endoplasmic reticulum, extracellular space, Golgi apparatus, lysosome, mitochondria, nucleus and peroxisome), in addition to intermembrane space of mitochondria with one metabolite (Fig. 3a). The fact that both models contain the same compartments is explained by their common origin in the human template Recon [32, 38]. Metabolites in the *E. siliculosus* reference model are mainly contained in the cytosol and extracellular space, while the remaining 4 compartments (mitochondria, periplasm, peroxisome and vacuole) contain 1 or 2 metabolites each (Fig. 3a). In all reference models, approximately half of duplicated reactions lack GPRs, while duplicated reactions with GPR are evenly divided into those having the same or different GPRs than their cytosolic counterparts (Fig. 3b and c).

AuReMe and merlin are the only methods that capture extensive eukaryotic compartmentalization. merlin models have 6–7 compartments (Fig. 3a) and the largest number of duplicated reactions, most of which have the same gene associations as their cytosolic counterparts (Fig. 3b). Notably, merlin includes a chloroplast compartment in the *E. siliculosus* model with a considerable size (629 metabolites, Fig. 3a). This compartment is also included by merlin in the *C. griseus* model, but it has 4 metabolites and could be easily corrected by manual curation. Although AuReMe models also display a high number of compartments (6–10, Fig. 3a) the presence of duplicated reactions is much lower than merlin, which is consistent with the smaller size of non-cytosolic compartments in AuReMe models. AuReMe also captures the chloroplast compartment in the *E. siliculosus* model, reflecting its use of a photosynthetic organism as a template model.

Pathway Tools, CarveMe and Reconstructor models display a basic compartmentalization comprising cytosol and extracellular space, as well as periplasm in the case of CarveMe, reflecting its original purpose as a tool for reconstructing bacterial GEMs (Fig. 3a). Consistent with this low level of compartmentalization, the three tools show relatively low reaction duplication, which is negligible for Reconstructor (Fig. 3c). Most duplicated reactions in Pathway Tools models have the same GPRs as their cytosolic counterparts while most duplicated reactions in CarveMe models have different GPRs than their cytosolic counterparts (Fig. 3c).

### Draft gene contents differ with respect to their references

**Fig. 4:**
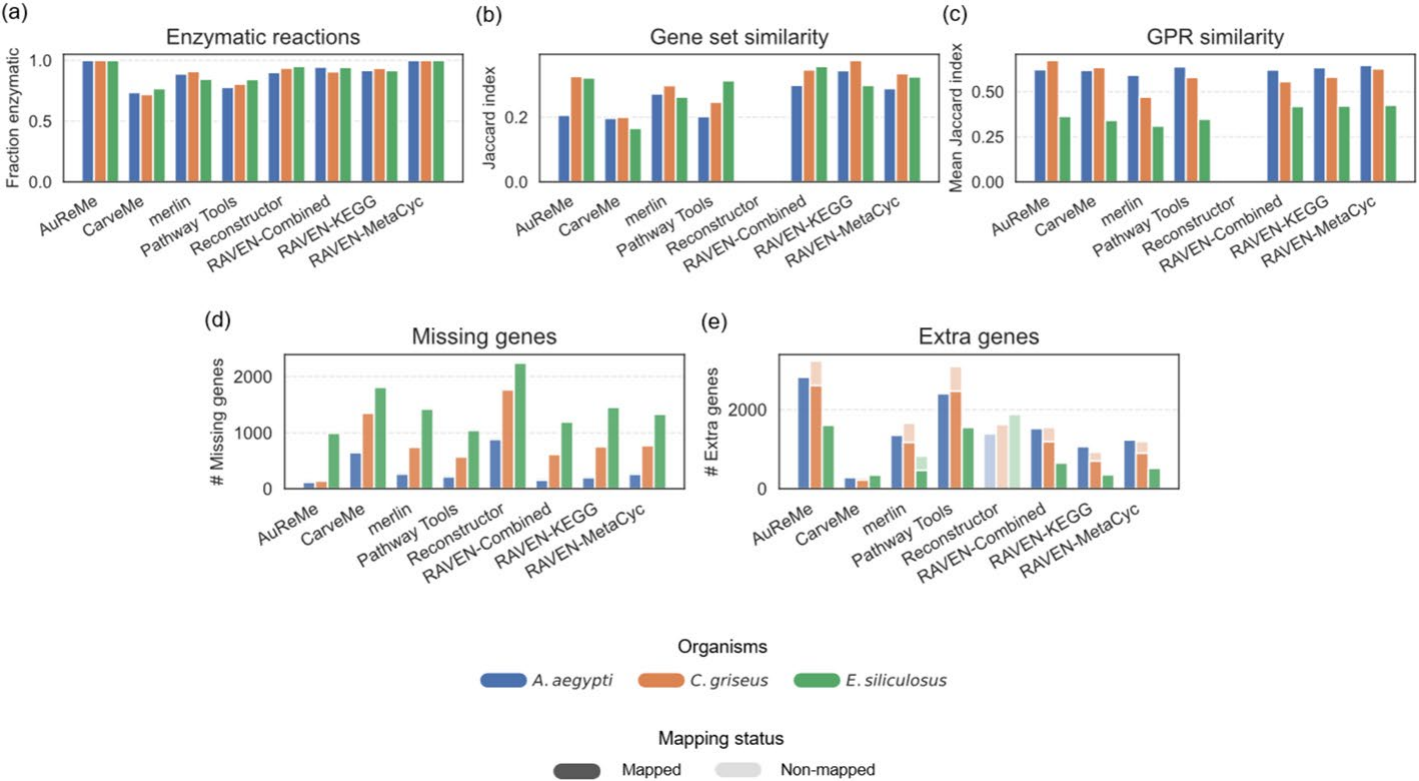
Genomic support of draft models. **a** Fraction of reactions with non-null GPRs. **b** Jaccard index between draft and reference gene sets. **c** Mean Jaccard index between the set of enzyme complexes for corresponding enzymatic reactions in reference and draft models (**d**, **e**) Missing and extra genes in draft models with respect to their references, distinguished by their mapping status (opaque and translucent bars denote genes successfully and unsuccessfully mapped to a common namespace, respectively)

GEM applications such as omics integration or simulation of genetic interventions benefit from genome-wide associations between reactions and the genes responsible for their catalysis. Furthermore, GPRs should correctly represent the logical dependence of reactions for genes or gene combinations, capturing the existence of isozymes or enzyme complexes. For each draft model, these traits were measured by the fraction of reactions with GPRs (Enzymatic reactions, Fig. 4a), the Jaccard index between draft and reference gene sets (Gene set similarity, Fig. 4b) and the mean Jaccard index between GPRs represented as sets of enzymatic complexes, across common enzymatic reactions in draft and reference (GPR similarity, Fig. 4c). The number of missing and extra genes in draft models with respect to their references (Fig. 4d, e) are also presented to assess gene set discrepancies.

CarveMe draft models exhibited the lowest proportions of reactions with associated GPRs (72–77%, Fig. 4a), which can be attributed to the extensive gap-filling strategy implemented by this method. In contrast, AuReMe and RAVEN-MetaCyc achieved 100% enzymatic reactions, reflecting a strict reliance on genome annotation for reaction inclusion. This conservative behavior is consistent with the low fractions of flux reactions observed for these tools (Fig. 1e) and their limited ability to produce biomass precursors in simulation (Fig. 2), highlighting a trade-off between genomic support and functional completeness.

Reconstructor draft models also showed high fractions of enzymatic reactions (> 90%), but exhibited null similarity with respect to reference gene sets and GPRs (Fig. 4b, c). This discrepancy arises because Reconstructor assigns genes from organisms other than the target reference species. As a result, Reconstructor models are poorly suited for applications that rely on species-specific GPRs, such as transcriptomic integration, unless additional orthology mapping steps are performed. Owing to the lack of gene comparability, Reconstructor results are excluded from the remaining analyses of Fig. 4.

Across organisms, gene set similarities between draft and reference models were modest (Fig. 4b), with Jaccard indices ranging from 0.17 to 0.38. These similarities are notably lower than those reported for prokaryotic reconstructions, where comparisons between draft and reference models of *Lactobacillus plantarum* and *Bordetella pertussis* yielded Jaccard indices between 0.38 and 0.62 [18]. Importantly, low gene set similarities were not driven by deficiencies in identifier mapping to the common NCBI namespace, as reference models achieved mapping rates above 96% and draft models showed an average mapping rate of 95% (Table S6). Instead, discrepancies are explained by large numbers of both missing and extra genes (Fig. 4d, e).

Missing genes (those present in reference models but absent from drafts) likely reflect the effects of manual curation in reference GEMs. For instance, the *C. griseus* reference model explicitly incorporated genes with relatively low homology to human metabolic genes to recover known metabolic capabilities [32], while extensive manual annotation of transporters was performed during the reconstruction of AraGEM [88], on which the *E. siliculosus* reference model is based [31]. Such curated associations may be absent from the annotation files used as input by some methods (e.g. AuReMe and Pathway Tools) or may not be recovered by automated annotation pipelines. In contrast, *A. aegypti* draft models exhibit the lowest number of missing genes, consistent with the fact that its reference GEM was largely built through orthology inference with the human proteome [38]. Genes included in this reference are therefore likely to correspond to well-characterized human orthologs, widely represented in the databases consulted by automatic reconstruction methods.

Extra genes (those present in draft models but absent from references) are more difficult to interpret, as reference models are expected to be comparatively comprehensive due to manual curation. One contributing factor may be the temporal gap between reference model construction and current draft reconstructions. The *A. aegypti* and *C. griseus* reference GEMs [32, 38] rely heavily on human Recon models published in 2016 or earlier [89–91], while the *E. siliculosus* reference model [31] is based on an *Arabidopsis thaliana* model from 2010 [88]. Since then, substantial advances in genome sequencing, annotation quality and metabolic database coverage may have enabled these methods to recover gene–reaction associations that were unavailable at the time of reference reconstruction.

To examine whether our conclusions depend strongly on the choice of reference model, we performed an additional comparison using iCHO3K, a recently proposed community-consensus reconstruction for Chinese hamster ovary cells (currently available as a preprint [92]). Automated draft models recovered 72–78% of iCHO3K genes, with Jaccard similarities of 0.4–0.5, compared to 0.3–0.4 when using the original CHO reference. As comparable community-consensus reconstructions are not yet available for *A. aegypti* or *E. siliculosus*, the original reference models were retained to ensure consistent cross-organism comparisons.

Even after accounting for this temporal effect, gene contents vary markedly across methods for draft reconstruction. Upon Principal Coordinate Analysis of gene sets (Figure S7), a consistent clustering pattern is observed, with RAVEN models grouping together (RAVEN-MetaCyc, RAVEN-KEGG and RAVEN-Combined), Pathway Tools-dependent reconstructions forming a second cluster (AuReMe and Pathway Tools), and the remaining methods (merlin and CarveMe) appearing more scattered. This indicates that despite starting from similar genomic inputs (proteomes or genome annotations) at a given time, methods arrive at different conclusions regarding gene inclusion in draft models, which could respond to differential presence of biochemical reactions in the databases that each method consults or in their associated GPRs. Notably, Pathway Tools and AuReMe exhibited the largest numbers of extra genes, suggesting that their annotation and pathway inference strategies may be more permissive, potentially favoring sensitivity over specificity in gene assignment.

More broadly, lower gene set similarities observed here compared to prokaryotic studies may reflect intrinsic features of eukaryotic genomes, including their larger size and complexity [33, 34], and the increased uncertainty these characteristics impose on genome annotation and orthology inference. Since gene identification constitutes the foundation for reaction inference, discrepancies at the gene level are expected to propagate to reaction and metabolite contents, which will be explored in the following section.

Beyond gene content alone, we evaluated how well draft models reproduce the logical structure of reference GPRs by comparing enzyme complexes for shared enzymatic reactions (Fig. 4c). *E. siliculosus* draft models displayed the lowest GPR similarities (mean Jaccard indices 0.31–0.43), which can be explained by the progress of biochemical knowledge in time and structural differences in GPR formulation as reference GPRs for this organism lacks AND operators, whereas draft models frequently include them (Table S8). For *A. aegypti*, all studied methods performed comparably, with mean GPR similarities ranging from 0.59 to 0.65. In contrast, *C. griseus* exhibited a broader spread (0.47–0.67), with merlin and AuReMe representing the lowest and highest performers, respectively. Importantly, differences in GPRs often reflect subset relationships rather than conflicting assignments (Table S8). Overall, GPR similarities were moderate to high, indicating that once a reaction is recognized as genome-associated by both a draft and a reference model, gene assignments tend to be relatively consistent.

Despite this agreement in GPR content, draft and reference models do not always coincide on whether a shared reaction is enzymatic. This is reflected in Matthews Correlation Coefficients below 0.41 across methods (Table S2). The lowest values correspond to comparisons involving models with 100% enzymatic reactions (AuReMe and RAVEN-MetaCyc) against references that include reactions without genomic support, whereas higher values (most notably for Pathway Tools) indicate partial concordance in identifying reactions lacking genomic backing in both draft and reference models.

### Databases influence draft metabolic composition rather than phylogeny

**Fig. 5:**
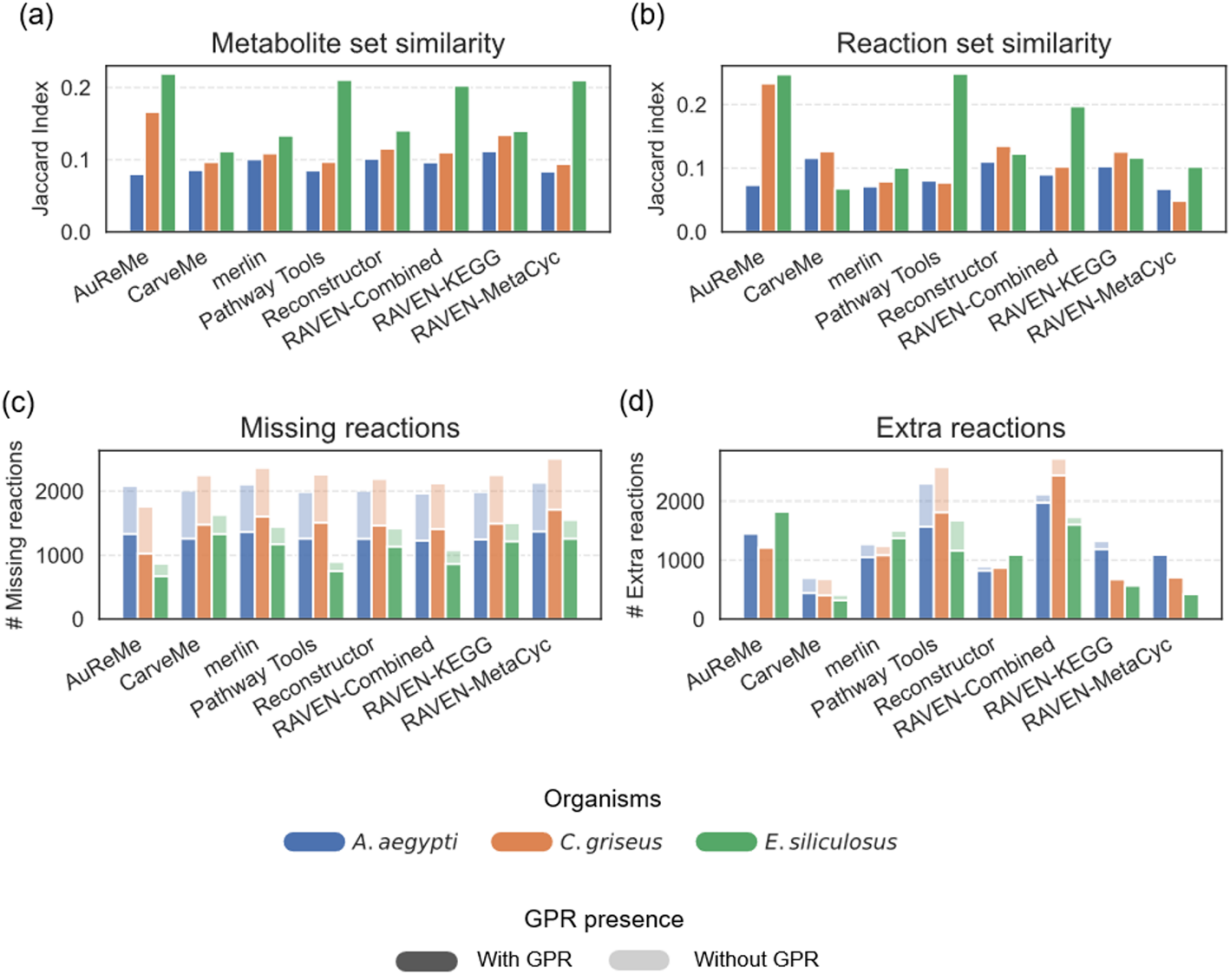
Similarity of draft biochemical networks with respect to references. **a** Jaccard index between draft and reference metabolite sets, after mapping to the MetaNetX namespace. **b** Jaccard index between draft and reference reaction sets, after removing transport reactions and mapping to the MetaNetX namespace. **c** Number of reactions present in references but not drafts, distinguished by GPR presence. **d** Number of reactions present in drafts but not references, distinguished by GPR presence

GEM applications benefit from biochemical networks accurately representing reactions and metabolites that can occur in an organism of interest, without over or underestimating its metabolic capabilities. Assuming accuracy of reference GEMs, metabolite and reaction sets from draft models should have similar elements compared to the references, captured by metrics Metabolite set similarity (Fig. 5a) and Reaction set similarity (Fig. 5b). Also, reactions present in the reference but not the draft (“missing reactions”) should ideally be those with low genomic support in the reference, while reactions present in the draft but not the reference (“extra reactions”) should have high genomic support in the draft. The number of missing and extra reactions and their genomic support is analyzed in Fig. 5c, d.

Metabolite and reaction set similarities ranged from 0.08 to 0.22 (Fig. 5a) and from 0.05 to 0.25 (Fig. 5b), respectively. AuReMe obtained the highest scores for *C. griseus* and *E. siliculosus*, the latter being comparable with Pathway Tools, while no method stood out for *A. aegypti* in terms of these metrics. Notably, metabolite and reaction set similarities were consistently lower than gene set similarities observed in Fig. 4b, a pattern that mirrors previous observations in prokaryotic benchmarks [18] and underscores the amplification of discrepancies as one moves from genes to reactions and metabolites. Low similarities cannot be attributed to mapping artifacts, as reactions and metabolites were mapped to the MetaNetX namespace with high success rates (> 96%, Table S9 and Table S10), nor to compartmentalization issues as transport reactions were omitted from the comparison and biochemically equivalent reactions from different compartments were mapped to the same MetaNetX IDs.

Among organisms, *E. siliculosus* drafts generally exhibited higher reaction and metabolite set similarities, which was accompanied by lower numbers of missing and extra reactions (Fig. 5c, d). In contrast, for *A. aegypti* and *C. griseus*, low Jaccard indices were primarily driven by large numbers of missing reactions, typically close to 2000 across methods. Approximately one third of these missing reactions lacked GPRs in the reference models, suggesting that they were introduced through gap-filling or manual curation. The remaining missing reactions (those with GPRs in the reference models) can be partially attributed to missing genes, however, for most *A. aegypti* and *C. griseus* draft models, a large fraction of missing reactions with GPRs in the reference depend on genes that are fully captured in draft models (Fig. S11). This means that draft reconstruction tools acknowledge the existence of these genes and their role in metabolic processes, but not their sufficiency for performing specific reactions, either due to database structure or methodological choices (e.g. stringency for adding reactions based on gene presence).

Extra reactions were generally less numerous than missing ones for *A. aegypti* and *C. griseus*, with notable exceptions for Pathway Tools and RAVEN-Combined. Extra reactions tended to have very high genomic support (Fig. 5d), consistent with the genome-driven philosophy of most draft reconstruction methods when adding reactions. The few extra reactions without associated GPRs, mainly observed in Pathway Tools and CarveMe models, can be explained by the automatic gap-filling performed by these methods. On the other hand, extra reactions with GPRs are not fully explained by extra genes in draft models, as a considerable fraction of these reactions depend on genes that are fully captured in reference models (Fig. S12). As in the case of missing reactions, this hints at differences in gene-reaction associations for genes that are consensually recognized as metabolism-related, likely due to differences in database structure or methodological choices during reaction addition.

**Fig. 6:**
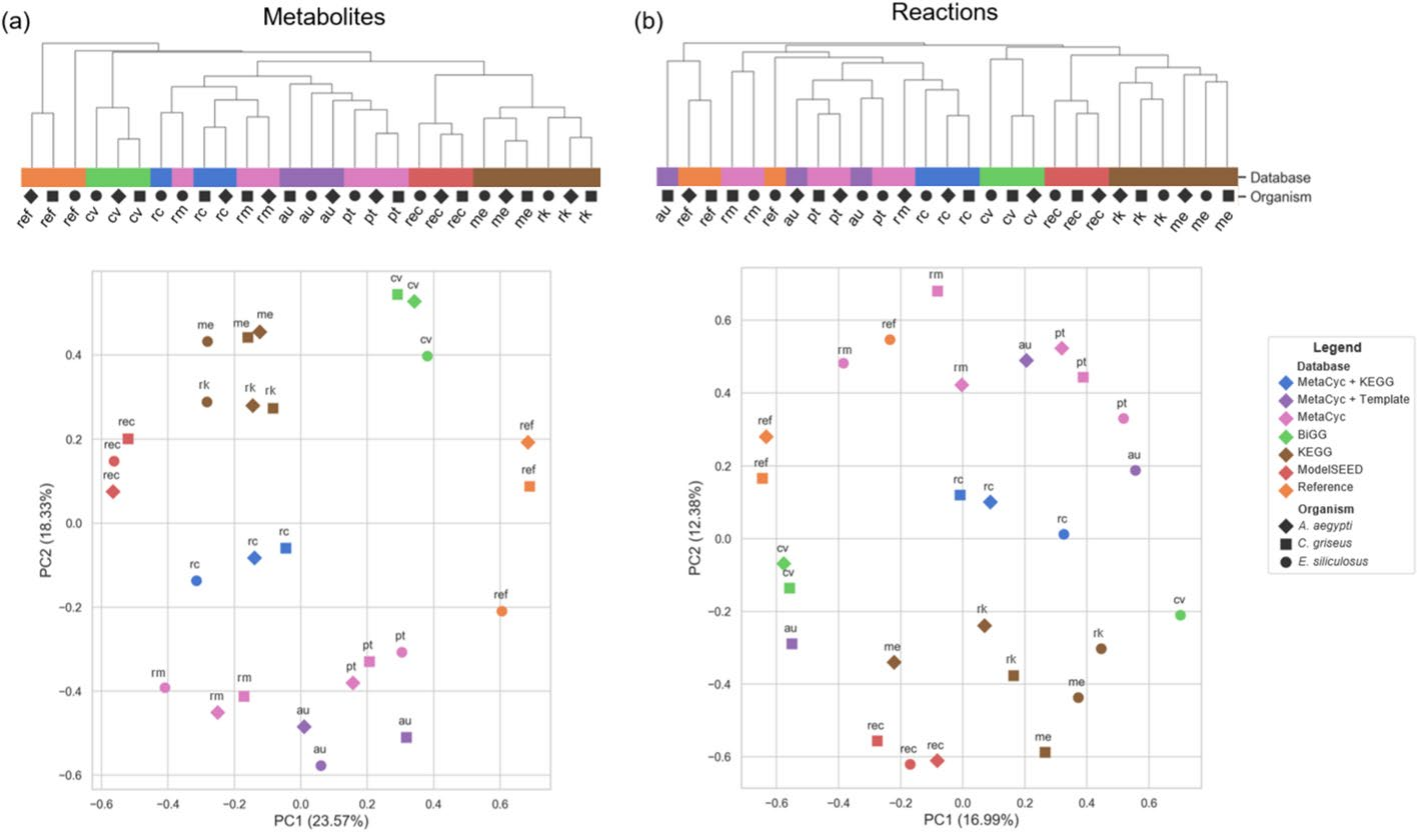
Influence of databases on metabolite and reaction inclusion in draft genome-scale models. Biclustering of reaction presence in draft models generated for this work, each method is presented with its associated database

To assess how reconstruction method and database choice shape draft model contents, metabolite and reaction sets were compared across all drafts (Fig. 6). As in the previous analysis (Fig. 5), metabolites and reactions were analyzed independently of compartment assignment. Under this representation, model similarity is driven mainly by reconstruction method and the database each method relies on, rather than by phylogeny.

Hierarchical clustering and PCoA shows that models group first by database and methods, and only within these groups does organism relatedness become relevant, with *A. aegypti* and *C. griseus* generally closer to each other than to *E. siliculosus* (Fig. 6a, b).

Metabolite content is primarily shaped by the reconstruction method, as shown by the clear method-specific clusters in Fig. 6a. Methods relying on KEGG (merlin and RAVEN-KEGG) and MetaCyc (Pathway Tools, RAVEN-MetaCyc and AuReMe) form distinct groups, while RAVEN-Combined models occupy an intermediate position, consistent with their mixed database basis.

For a given method, organism relatedness explains part of the remaining variability, with *A. aegypti* and *C. griseus* generally closer to each other than to *E. siliculosus*. Reaction sets show a similar pattern (Fig. 6b). AuReMe reaction models do not collapse into a single cluster, reflecting the use of different organism-specific templates. CarveMe models form a compact cluster, but the *E. siliculosus* model is distant from the animal models, consistent with the use of a universal bacterial reference.

By ignoring compartment assignments, this analysis focuses on biochemical content independently of subcellular localization. Under this representation, merlin and RAVEN-KEGG models cluster together (Fig. 6a, b), a pattern that reflects shared database content and would otherwise be dominated by differences in compartmentalization between methods.

**Fig. 7:**
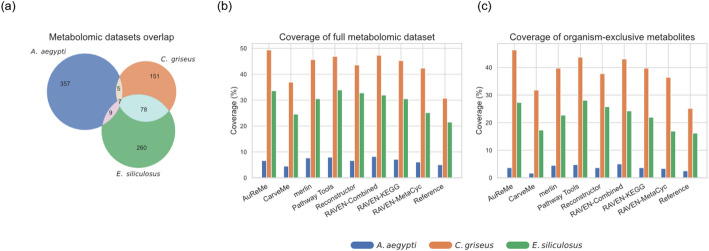
Metabolomic coverage analysis. **a** Metabolomic datasets retrieved from literature for *A. aegypti*,* C. griseus* and *E. siliculosus*. **b** Full metabolome coverage (**c**) Organism-specific metabolome coverage (i.e. coverage of metabolites that only appear for the corresponding organism)

Multicellular organisms produce specialized metabolites that are absent in prokaryotes, and most unicellular eukaryotes. To evaluate whether draft models capture these metabolic features, metabolomic sets were compiled for *A. aegypti* [75], CHO cells [32, 76–84] and *E. siliculosus* [85], and assessed their coverage by each model (Fig. 7).

Query metabolomic datasets for *A. aegypti*, *C. griseus* and *E. siliculosus* comprised 378, 241 and 354 metabolites, respectively. These datasets are mostly organism-specific, that is, their intersections are relatively small sharing only seven metabolites across all three organisms (histidine, proline, tryptophan, ornithine, thymidine, adenosine monophosphate and 1-oleoyl-glycerophosphoinositol; Fig. 7a). This explains the similar behavior between the coverage of full metabolomic datasets (Fig. 7b) and of exclusive metabolomic datasets (consisting of metabolites included for an organism but not the rest, Fig. 7c).

Across organisms, variability in metabolome coverage is not consistently associated with organism complexity or annotation depth. Instead, dispersion differs primarily across reconstruction methods, indicating that metabolite recovery is driven by methodological and database-related factors rather than by the choice of reference organism.

Metabolomic coverages of *A. aegypti* models are uniformly low across methods (Fig. 7), reflecting that the corresponding metabolomic dataset is enriched in metabolites that are intrinsically difficult to capture in GEMs (Table S13). In particular, it contains a high proportion of structurally diverse lipids and lipid conjugates with defined fatty-acyl chain lengths and degrees of unsaturation. In GEMs, lipid metabolism is commonly represented using lumped or generic lipid species rather than explicitly enumerating the large combinatorial diversity of fatty-acid chain compositions detected by lipidomics, meaning that many measured molecular species have no direct counterparts in the reconstruction even if the underlying biosynthetic pathways are present [93–95]. This limitation is further amplified by the structural diversity and enzyme specificity characteristic of many lipid subclasses, which are not always comprehensively represented in the biochemical resources typically used for automated model reconstruction. Our results highlight that targeted manual curation of lipid metabolism may be required, particularly when this subsystem is of research interest [94]. Such efforts can be supported by specialized lipid knowledgebases such as LIPID MAPS [96], which provides detailed lipid classification and molecular species information, and SwissLipids [97], which integrates lipid structures with curated metabolic reactions and enzyme annotations.

Reference models do not display the best coverage, which likely reflects differences in database content and versioning rather than reconstruction quality as observed previously. Among the evaluated methods, AuReMe consistently achieves the highest coverage of reported metabolites, whereas CarveMe models tend to recover the smallest subsets.

This behavior reflects the reconstruction approach used by each method. AuReMe incorporates organism-specific template integration, leading to higher metabolite coverage than Pathway Tools-derived drafts alone. In contrast, CarveMe builds from a universal bacterial model, which explains both its reduced model size (Fig. 1) and its limited recovery of organism-specific metabolites.

Previous comparisons of biochemical content (Fig. 5) relied on the assumption that curated reference models represent the closest approximation to an underlying metabolic truth. While useful, this approach inherently evaluates draft reconstructions within a closed reference-based framework. The metabolome-based analysis presented here breaks this loop by introducing independent empirical data, providing a complementary perspective on reconstruction performance that is not constrained by the content of existing GEMs.

## Discussion

Organism-specific reconstruction of GEMs is a complex process that can take up to years. This has motivated the development of automated tools that help accelerate this task. However, selecting a tool for draft generation remains non-trivial and usually depends on user expertise. An ideal model should accurately represent the metabolic landscape of the organism, requiring minimal manual curation, and supporting typical GEM applications such as flux simulations and omics data integration. In this work we assessed seven methods in terms of functionality, gene associations, biochemical accuracy and compartmentalization of their produced draft GEMs aiming to guide tool selection for eukaryotic GEM reconstruction.

Selected organisms for this comparison span different levels of curation and phylogenetic distance, from well-characterized mammalian cell lines to a multicellular algae with a more limited reference model. These organisms were chosen to represent contrasting biological contexts commonly encountered in genome-scale modeling studies.

Selected methods include tools originally developed for prokaryote organisms (CarveMe, Reconstructor), and PlantSEED, a plant-focused adaptation of the ModelSEED framework. Draft models generated using these approaches display biomass representations based on gram positive or negative bacteria, as well as a reduced number of compartments. These methods were included based on their prevalence for their comparison with methods more compatible with eukaryotes, and although they retrieve in some cases closely to functional models their predictions should be used with caution given the phylogenetic distance between the organisms for which they were developed for in contrast with the organisms analyzed in this work.

Other available methods, such as metaDraft [98] and gapseq [27], were considered for this comparison but were unable to retrieve any results for the analyzed organisms due mainly to technical issues associated with the large genomes of these organisms, and lack of organism-specific data in KEGG (AutoKEGGRec [20]). While these tools may be suitable for other contexts, their exclusion here reflects practical constraints rather than an assessment of their methodological quality.

A central result of this work is that variability in biochemical model content is driven primarily by the reconstruction method and its underlying database, rather than by the organism itself. This trend persists even after decompartmentalizing reactions and metabolites and excluding transport reactions, indicating that differences are not explained by the presence or absence of compartmentalization. Consequently, different reconstruction tools can generate GEMs with substantially different biochemical contents even when provided with the same input genome, highlighting methodological choice as a key determinant of model structure.

In addition to differences in biochemical content, reconstruction methods also exhibit marked variability in their ability to fulfill specific modeling tasks. For instance, tools developed for prokaryotes, such as CarveMe and Reconstructor, efficiently generate near-functional models but rely on simplified biomass formulations and limited compartmentalization, which constrain their applicability to eukaryotic systems. In contrast, approaches based on curated databases such as MetaCyc (e.g., Pathway Tools, RAVEN, AuReMe) can improve the representation of organism-specific pathways, although they often do not directly yield functional models. Methods that explicitly infer enzyme localization, such as merlin, enhance the representation of compartment-specific gene-protein-reaction associations, which is particularly relevant for integrating expression data into GEMs [9, 10].

These trade-offs extend to practical considerations such as execution time, required expertise, and suitability for different applications. For example, merlin produces highly compartmentalized and well-annotated models but requires substantial time and manual intervention, whereas CarveMe and Reconstructor are better suited for rapid, large-scale or exploratory analyses. AuReMe offers flexibility for incorporating prior knowledge in less-characterized organisms, albeit with a steep learning curve. A structured summary of these strengths, limitations, and recommended usage scenarios is provided in Table S14, which can serve as a practical guide for tool selection.

Given this variability across methods, consensus approaches that integrate the strengths of multiple draft reconstructions could provide a more comprehensive representation of organismal metabolism. While such approaches have been previously applied [98–102], they were focused on prokaryotes or were manual, leaving the development and benchmarking of automated frameworks for combining eukaryotic draft GEMs as an open challenge. In the meantime, manual integration efforts may benefit from using compartmentalized reconstructions as templates, as they better capture transport processes and the spatial organization characteristic of eukaryotic metabolism.

Curated reference models are commonly used as benchmarks for evaluating draft reconstructions; however, our results indicate that they should not be regarded as absolute gold standards. Reference GEMs exhibited heterogeneous annotation quality, suboptimal consistency metrics, and incomplete representation of enzyme complexes in the case of *E. siliculosus*, which limits their reliability as a definitive ground truth for model evaluation.

In this context, manual curation and expert knowledge remain essential for obtaining high-quality GEMs. The absence of a well-defined gold standard implies that model validation should rely on multiple complementary sources of evidence, including orthogonal omics datasets (e.g., metabolomics), phenotypic measurements (e.g., growth, secretion, or uptake rates), and prior knowledge of specific metabolic pathways, ideally guided by domain expertise. Our results further highlight specific areas where manual intervention is particularly important. For example, all evaluated methods showed limited ability to capture structurally diverse lipid species supported by metabolomic data, indicating that lipid metabolism may require targeted curation when it is central to the study. Additionally, manual refinement is often necessary to correct systematic biases of reconstruction tools originally developed for prokaryotes, such as assumptions regarding biomass composition.

Systems biology is an interdisciplinary area of research that merges people with different backgrounds. This heterogeneity results in applications of genome-scale models from exploration of metabolic capabilities [31], to biotechnological applications [32, 103, 104] and even analysis with ecological implications [105]. Hence, the need for tools with different approaches designed with different applications in mind. Our results provide a systematic comparison that clarifies the strengths and limitations of current approaches, supporting more informed tool selection (as summarized in Table S14), and highlights key directions for future methodological development in the reconstruction of genome-scale metabolic models for multicellular eukaryotes.

## Supplementary Information

Below is the link to the electronic supplementary material.

Supplementary Material 1

Supplementary Material 2

Supplementary Material 3

Supplementary Material 4

Supplementary Material 5

Supplementary Material 6

Supplementary Material 7

Supplementary Material 8

Supplementary Material 9

Supplementary Material 10

Supplementary Material 11

Supplementary Material 12

Supplementary Material 13

Supplementary Material 14

Supplementary Material 15

## Data Availability

All genome and annotation files used in this study are publicly available. The *Cricetulus griseus* genome (CriGri_1.0) and its annotation were retrieved from NCBI under the accession GCA_000223135.1. The *Aedes aegypti* genome (AaegL5.0) and annotation were obtained from VectorBase (Matthews et al., 2018). The *Ectocarpus siliculosus* genome (ASM31002v1) and annotation were retrieved from NCBI under the accession GCA_000310025.1. Reference genomes used for orthology-based reconstructions, including *Drosophila melanogaster* (GCF_000001215.4), Mus musculus (GCF_000001635.27), *Chlamydomonas reinhardtii* (GCF_000002595.2), *Homo sapiens* (GCF_000001405.40), and *Saccharina japonica*; were also obtained from NCBI and relevant databases, as cited in the manuscript. Data (obtained models as well as their analysis) is provided within the manuscript, supplementary information files or the github repository [https://github.com/natJimenez/eukaryo_methods].
