# Supplementary material for: Selecting methods for draft GEM generation in multicellular eukaryotes: a comparative analysis
Source: BMC Bioinformatics. 2026 May 22;27:153. doi: 10.1186/s12859-026-06455-7 (PMC13390297; doi:10.1186/s12859-026-06455-7)
Supplement: Supplementary file 1 — Supplementary Material 1 [file 12859_2026_6455_MOESM1_ESM.docx]

**Table S1: Summary of methods analyzed in this work**

| Tool | Inputs | Outputs | Reference |
| --- | --- | --- | --- |
| AuReMe | **Annotation-based reconstruction:** Pathway Tools draft model (.dat folder)  **Orthology-based reconstruction:** Proteome of both target organism and template organism (.faa). Genome-scale model of template organism (.sbml) | Draft genome-scale model | [[28]](https://www.zotero.org/google-docs/?Txgdft) |
| CarveMe | Proteome of target organism (.faa), taxonomy of target organism. | Functional genome-scale model (.sbml), with predefined biomass function based on parameter selection. | [[24]](https://www.zotero.org/google-docs/?Dn5LX5) |
| merlin | Proteome of target organism (.faa) | Draft genome-scale model (.sbml), visualization of its metabolic pathways. | [[22]](https://www.zotero.org/google-docs/?knMjlg) |
| PlantSEED | Proteome of target organism (.faa), taxonomy of target organism. | Functional genome-scale model (.sbml) with predefined biomass based on taxonomy. | [[25]](https://www.zotero.org/google-docs/?25Jw7J) |
| Pathway Tools | Annotated genome (.gbk) | Database of metabolic pathways, reactions and metabolites (.dat files). Draft genome-scale model (.sbml). Visualization of its metabolic pathways. | [[23]](https://www.zotero.org/google-docs/?54s8rb) |
| RAVEN 2 | Proteome of target organism (.faa) | Draft genome-scale model (.sbml) based on KEGG, MetaCyc databases or both. | [[21]](https://www.zotero.org/google-docs/?pVLfM5) |
| Reconstructor | Proteome of target organism (.faa), taxonomy of target organism (user input). | Draft genome-scale model (.sbml) | [[19]](https://www.zotero.org/google-docs/?zKaEqz) |
