# Supplementary material for: Selecting methods for draft GEM generation in multicellular eukaryotes: a comparative analysis
Source: BMC Bioinformatics. 2026 May 22;27:153. doi: 10.1186/s12859-026-06455-7 (PMC13390297; doi:10.1186/s12859-026-06455-7)
Supplement: Supplementary file 3 — Supplementary Material 3 [file 12859_2026_6455_MOESM3_ESM.docx]

**S4 - Effect of template model in AuReMe**

**Further analysis with alternative template models using this tool showed that predictions made from orthology exhibit a trade off between phylogenetic closeness and the information contained in models used for their reconstruction (Figure S4.1). Phylogenetic distance within modeled organisms is a key differentiating factor at a metabolic level for both metabolite and reaction content included in the analyzed drafts, where models for *E. siliculosus* are grouped together at both analyzed levels with the exception of the draft retrieved from orthology between *E. siliculosus* and *C. reinhardtii*. This model retrieves reactions linked to the orthology step for our original template selected for this organism, a microalgae which could explain their behaviour at both levels. On the other hand, template models from phylogenetically close organisms *(Mus musculus* and *Homo sapiens* for *C. griseus* draft generation) retrieve almost identical models at a metabolite level and similar models at a reaction level.**

**
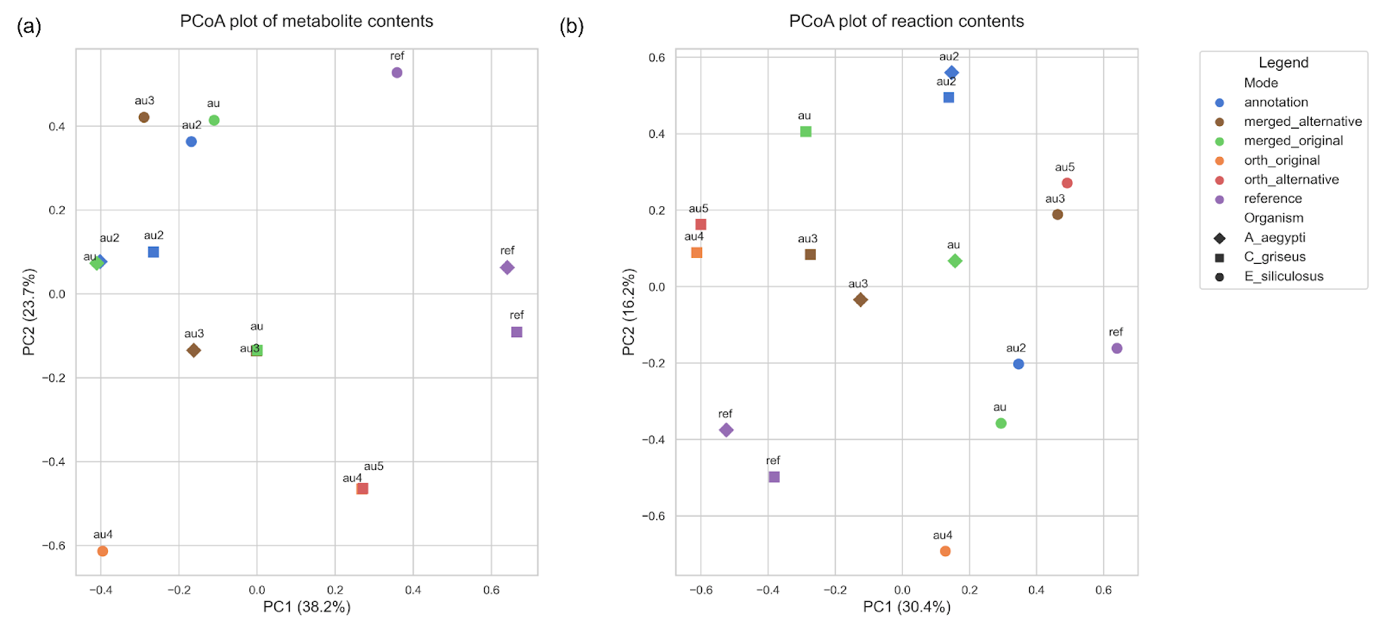
**

**Figure S4.1: Effect of different inputs and stages of generation in AuReMe draft generation models on metabolite (a) and reaction (b) contents. From annotation steps performed by Pathway tools (annotation), orthology steps performed using original templates (orth_original) as well as alternative templates (orth_alternative) as specified in methods; to finally, merged models using both approaches (merged_original, merged_alternative). Reference models are included to depict their similarity to obtained draft models.**

**Similarities between different stages of draft generation using the AuReMe workflow (annotation, orthology, merged) are observed. For *Ectocarpus*, merged and annotation models from original template are more similar with each other than with orthology template, alternative template (*S. japonica*) retrieves a model which is closer with these models, hinting a possible database-specific effect. For *Cricetulus* and *Aedes* the effect of the alternative template was strikingly clear, with the *Aedes* alternative draft model (template: *H. sapiens*) was more similar to the *C. griseus* draft based on the same template model.**
