# Supplementary figures and images for: Selecting methods for draft GEM generation in multicellular eukaryotes: a comparative analysis
Source: BMC Bioinformatics. 2026 May 22;27:153. doi: 10.1186/s12859-026-06455-7 (PMC13390297; doi:10.1186/s12859-026-06455-7)

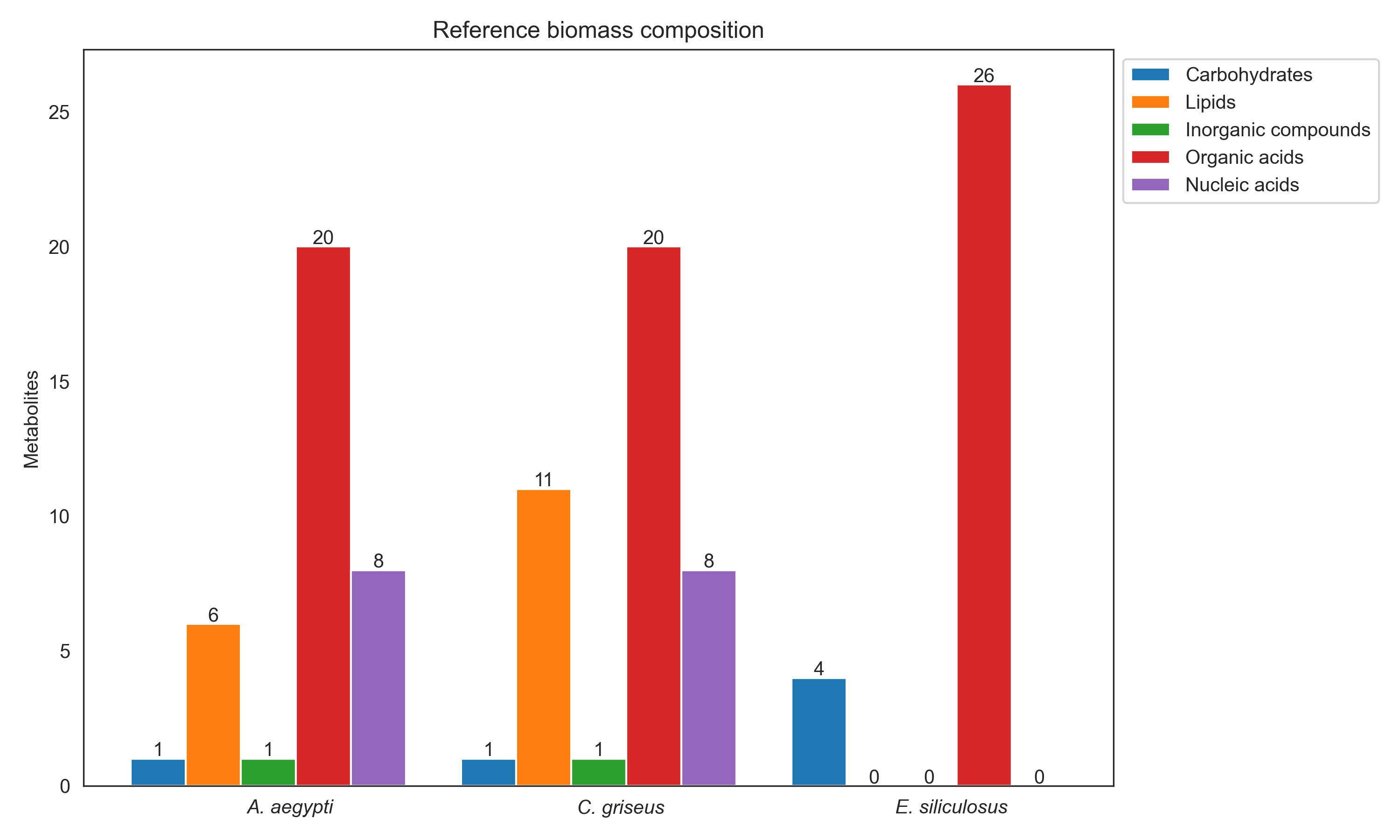

Supplement: Supplementary file 7 — Supplementary Material 7 [file 12859_2026_6455_MOESM7_ESM.png]
