# Supplementary material for: Selecting methods for draft GEM generation in multicellular eukaryotes: a comparative analysis
Source: BMC Bioinformatics. 2026 May 22;27:153. doi: 10.1186/s12859-026-06455-7 (PMC13390297; doi:10.1186/s12859-026-06455-7)
Supplement: Supplementary file 14 — Supplementary Material 14 [file 12859_2026_6455_MOESM14_ESM.docx]

# **Table S14:** Summary of strengths and limitations of each method

| Method | Strengths | Limitations | Suitable applications |
| --- | --- | --- | --- |
| AuReMe | - Represents eukaryotic compartmentalization, including photosynthetic compartments.  - Complete representation of biomass precursors.  - High recovery of extra genes and enzymatic reactions, and low number of missing genes. | - Requires a template model of a phylogenetically close organism.  - No producibility of biomass precursors. - Low fraction of flux reactions. - Steep learning curve. | - Study of non-model organisms, provided a phylogenetically close template exists.  - Study of eukaryotic metabolism, including transport-limited or compartment-specific metabolism.  - Initial draft generation for long-term comprehensive modeling.  - Flux simulations, including simulation of genetic interventions or omics-constrained metabolism after significant gap-filling. |
| CarveMe | - High fraction of flux reactions.  - Producibility of biomass carbohydrates and nucleic acids.  - Fastest model reconstruction. | - No representation of lipids nor producibility of organic acids (despite being represented).  - Relies on a universal bacterial model.  - Bacterial biomass assumptions  - Bacterial compartmentalization | - Flux simulations involving core metabolism and no transport limitation.  - Simulation of genetic intervention effects for core reactions, without requiring additional gap-filling.  - Rapid hypothesis generation and testing. |
| merlin | - Represents eukaryotic compartmentalization, including photosynthetic compartments*.  - Complete representation of biomass precursors.  - Producibility of biomass nucleic and organic acids.  - Highest annotation score.  - Considerable inclusion of extra genes and enzymatic reactions with respect to reference. | - Requires multiple manual steps resulting in long execution times.  - No producibility of biomass carbohydrates or lipids. | - Study of eukaryotic metabolism, including transport-limited or compartment-specific metabolism, especially in absence of a template model.  - Flux simulation, including simulation of genetic interventions or omics-constrained metabolism after gap-filling. - Models for transcriptomic/proteome integration  - Initial draft generation for long-term comprehensive modeling. |
| Pathway Tools | - Organism-specific pathways - Metacyc-based curation  - High gene recovery  - Complete representation of biomass precursors.  - Producibility of biomass carbohydrates and lipids. | - Models often not functional - Requires manual curation (no producibility of biomass nucleic or organic acids).  - No compartmentalization.  - Considerable fraction of extra reactions without gene association. | - Biochemical pathway exploration  - Flux simulations targeted at studying lipid metabolism, after significant gap-filling.  - Complementing compartmentalized draft models, upon long-term comprehensive modeling. |
| PlantSEED | - Curated pathway content tailored to plant primary metabolism  - Easy to use (web user interface) | - Biochemical networks are insensitive to organism-specific input data, due to aggressive gap-filling. | - Exploration of plant primary metabolism  - Exploration of reactions included based only on gene-based information |
| RAVEN (MetaCyc, KEGG and  Combined) | - Access to multiple curated databases.  - Complete representation of biomass precursors.  - Producibility of biomass nucleic and organic acids in KEGG and Combined mode. Biomass lipid producibility in KEGG mode.  - High percentage of reactions associated with genes (100% for MetaCyc mode).  - Considerable recovery of extra genes and reactions with respect to reference, especially in the Combined mode. | - Low fraction of flux reactions.  - No producibility of biomass precursors in MetaCyc mode. No producibility of nucleic acids in Combined mode.  - No compartmentalization. | - Draft reconstruction for diverse organisms  - Complementing compartmentalized draft models, upon long-term comprehensive modeling. |
| Reconstructor | - Faster than most methods - Close to functional drafts  - Producibility of biomass nucleic and organic acids. | - Gene identifiers are not from the modeled organism, requiring ortholog mapping for model applications relying on gene annotations.  - No producibility of biomass carbohydrates or lipids.  - No compartmentalization. | - Expansion of known pathways supported by wide orthology exploration.  - Complementing compartmentalized draft models, upon long-term comprehensive modeling. |

# * merlin included a plastid compartment for *A. aegypti* which, however, is of minimal size and can be easily corrected by manual curation.
